# Structural investigations of proteins encoded by SARS‐CoV‐2

**DOI:** 10.1002/2211-5463.13465

**Published:** 2022-08-31

**Authors:** Alexander Wlodawer

**Affiliations:** ^1^ Center for Structural Biology, Center for Cancer Research National Cancer Institute Frederick MD USA

**Keywords:** coronavirus, covid, protein structure, ribonuclease, SARS‐CoV‐2, spike protein

## Abstract

It is hard to overestimate the influence of the COVID‐19 pandemic on scientific research in the last two and a half years. Within a few weeks after the first cases of the disease were reported, the causative agent, now known as SARS‐CoV‐2, was identified, its genome was sequenced, individual proteins were expressed and purified, and structural work commenced. The originally described SARS‐CoV‐2 isolate (GenBank: MN908947.3) has a positive‐sense single‐stranded (ss) RNA genome consisting of 29,903 bases. The genome encodes 29 proteins falling into structural and nonstructural categories, expressed as polyproteins that have to be cleaved into the final products by two virally encoded cysteine proteases. This “In the Limelight” special issue of *FEBS Open Bio* includes three review articles, focused on different aspects of the structure and other properties of selected examples of SARS‐CoV‐2 proteins: (a) the properties of the Nsp14 and Nsp15 ribonucleases; (b) the current state of knowledge of the molecular mechanisms for the translation of both viral transcripts and cellular messenger RNAs, with a focus on the properties of the Nsp1 protein; and (c) the structural properties and evolution of the spike proteins in SARS‐CoV‐2 and other coronaviruses. These three reviews describe very different aspects of work that ultimately should lead to the development of more vaccines, antibodies, and small molecule drugs, necessary to combat this pandemic, as well as to counter future variants of this coronavirus.

## Graphical abstract

This “In the Limelight” issue includes three review articles, focused on different aspects of the structure and other properties of certain SARS‐CoV‐2 proteins: (i) the Nsp14 and Nsp15 ribonucleases; (ii) molecular mechanisms for the translation of viral transcripts and cellular mRNAs, with a focus on Nsp1; and (iii) the structural properties and evolution of spike proteins in SARS‐CoV‐2 and other coronaviruses.

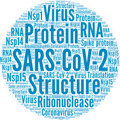

**Abbreviations**
MTase: methyltransferase
PDB: Protein Data Bank
ssRNA: single‐stranded RNA

It is hard to overestimate the influence of the COVID‐19 pandemic on scientific research in the last two and a half years. Within a few weeks after the first cases of the disease were reported, the causative agent, now known as SARS‐CoV‐2, was identified, its genome was sequenced, individual proteins were expressed and purified, and structural work commenced. Of course, structural investigations represented only a small fraction of all work on SARS‐CoV‐2, the extent of which has been without precedent. A search of titles of scientific publications on PubMed that contain the phrases “SARS‐CoV‐2” or “COVID‐19” yielded over 225,000 hits (as of June 25, 2022). An equivalent search of depositions in the Protein Data Bank (PDB) yielded almost 2100 structures. By comparison, 37 years of research on HIV‐1/AIDS yielded almost 280,000 papers and 2800 structures. There is no question that the rate of progress of COVID‐19 studies has been unparalleled, already leading to the creation of several vaccines, therapeutic antibodies, and small molecule drugs.

The originally described SARS‐CoV‐2 isolate (GenBank: MN908947.3) has a positive‐sense single‐stranded (ss) RNA genome consisting of 29,903 bases. The genome encodes 29 proteins falling into structural and nonstructural categories, expressed as polyproteins that have to be cleaved into the final products by two virally encoded cysteine proteases. The majority of the viral proteins have been extensively characterized in both biochemical and structural terms. In addition, host proteins that interact with the viral proteins have also been extensively investigated. Obviously, it would be impossible to summarize all that has been learned about them in a single journal issue. Thus, this “In the Limelight” special issue of *FEBS Open Bio* covers only a very small fraction of what has been learned about the structure and other properties of selected examples of SARS‐CoV‐2 proteins.

Robin Stanley kand her colleagues review the properties of two ribonucleases that process the viral RNA [1]. Nsp14 is an exonuclease, whereas Nsp15 is an endonuclease. Nsp14 consists of an N‐terminal exonuclease domain and a C‐terminal N7‐methyltransferase (MTase) domain. The ribonuclease domain of Nsp14 plays a very important role in viral replication, by engaging in proofreading and maintaining the integrity of the viral genome. Nsp15 is a uridine‐specific endoribonuclease comprised of the N‐terminal, middle, and catalytic domain. This enzyme forms a hexamer that is required to maintain its activity. Nsp15 nuclease activity is important in the evasion of the host immune response to the virus. Both Nsp14 and Nsp15 have been extensively characterized, and their structural and mechanistic properties are discussed in considerable detail.

A review by Eriani and Martin summarizes the current state of knowledge of the molecular mechanisms for the translation of both viral transcripts and cellular messenger RNAs [2]. It concentrates on the properties of Nsp1, a protein that binds to ribosomes and stimulates translation termination of cellular mRNAs. This amino acid sequence of this protein is very highly conserved in different strains of SARS‐CoV‐2. Several crystal structures of Nsp1 are present in the PDB, but are not discussed in detail in this review. However, the role of Nsp1 in other coronaviruses that infect not only humans, but also some economically very important animals (such as pigs and poultry) is covered.

It is impossible to overestimate the importance of the knowledge gained on the structural properties of the spike protein of coronaviruses. The SARS‐CoV‐2 surface spike glycoprotein mediates receptor binding and membrane fusion for cell entry, playing critical roles in the process of infection and evolution. A review by Xinquan Wang and his coworkers describes the structural properties and evolution of the spike proteins in SARS‐CoV‐2 and other coronaviruses, as well as recent progress in developing antibodies, nanobodies, and peptidic inhibitors that could be used for therapeutic purposes [3].

These three reviews describe very different aspects of work that ultimately should lead to the development of more vaccines, antibodies, and small molecule drugs, necessary to combat this pandemic, as well as to counter future variants of this coronavirus that caused major disruption to economic activity in almost all countries. They provide just a flavor of the importance of such research, but can be used as a starting point in learning about many aspects of structure and function of proteins and nucleic acids that are components of SARS‐CoV‐2, viruses that for a while paralyzed the world.

## Conflict of interest

The author declares no conflict of interest.

## Author contributions

AW wrote the article.
